# Establishment of A Reversibly Inducible Porcine Granulosa Cell Line

**DOI:** 10.3390/cells9010156

**Published:** 2020-01-08

**Authors:** Yinshan Bai, Cui Zhu, Meiying Feng, Bo Pan, Shouquan Zhang, Xiaoshu Zhan, Huifang Chen, Bingyun Wang, Julang Li

**Affiliations:** 1 School of Life Science and Engineering, Foshan University, Foshan 528231, China; xuefei200403@163.com (Y.B.); juncy2010@gmail.com (C.Z.); chenhuifang07@163.com (H.C.); bywang63@163.com (B.W.); 2 Department of Animal Biosciences, University of Guelph, Guelph, ON N1G 2W1, Canada; bopan@uoguelph.ca (B.P.); xzhan01@uoguelph.ca (X.Z.); 3 College of Animal Science, South China Agricultural University, Guangzhou 510642, China; jony.ya@163.com (M.F.); sqzhang@scau.edu.cn (S.Z.)

**Keywords:** granulosa cells, Tet-on 3G, Large T, proliferation, immortalization, steroidogenesis

## Abstract

Granulosa cells (GCs) are the key components of ovarian follicles for regulating oocyte maturation. Previous established GC lines have allowed prolonged proliferation, but lost some physiological features owing to long-term immortalization. This study was to establish an induced immortal porcine GC line with reversible proliferation status by the tetracycline inducible (Tet-on) 3G system. Our conditional immortal porcine GCs (CIPGCs) line steadily propagated for at least six months and displayed primary GC morphology when cultured in the presence of 50 ng/mL doxycycline [Dox (+)]. Upon Dox withdrawal [Dox (–)], Large T-antigen expression, reflected by mCherry fluorescence, gradually became undetectable within 48 h, accompanied by less proliferation and size increase. The levels of estradiol and progesterone, and the expression of genes associated with steroid production, such as *CYP11A1* (cytochrome P450 family 11), *3β-HSD* (3β-hydroxysteroid dehydrogenase), *StAR* (steroidogenic acute regulatory protein), and *CYP19A1* (cytochrome P450 family 19 subfamily a member 1), were all significantly higher in the Dox (–) group than Dox (+) group. The CIPGCs could switch into a proliferative state upon Dox induction. Interestingly, the expression of *StAR* and *CYP19A1* in the CIPGCs (–Dox) was significantly increased by adding porcine follicular fluid (PFF) to mimic an ovary follicle environment. Moreover, PFF priming the CIPGCs in Dox (–) group resulted in similar estradiol production as that of primary GC, and enabled this cell line to respond to gonadotrophins in estradiol production. Collectively, we have established an inducible immortal porcine GC line, which offers a unique and valuable model for future research on the regulation of ovarian functions.

## 1. Introduction

Granulosa cells (GCs) play important roles in oocyte development, ovulation, and pregnancy [1]. Granulosa cell culture in vitro is a valuable model for studying female reproductive hormone synthesis, follicular development, and fertility [2]. However, culture of primary GCs has been difficult owing to their limited numbers and life span in vitro, as well as their quick luteinization within a short period of time after isolation [3], which have restrained their research potential in vitro.

Previous studies have reported that GCs can be immortalized by transfecting genes such as Simian virus 40 Large T-antigen (Large T) [4,5] or telomerase reverse transcriptase (Tert) [6]. This ability is because of their inhibition on cell cycle suppressors (p53 and pRb), cell proliferation enhancing (Large T), or telomer extension (Tert) (reviewed in the work of [7]). The immortalized GC lines from various species have been previously established by expression of Large T and Tert [4,5,8] or spontaneous immortalization [9]. These GC lines can proliferate in vitro and retain some functional characteristics of primary GCs [10]. However, many of the key physiological functions are lost in GC lines owing to permanent cellular transforming [4,9,10,11]. For example, several porcine GC lines, including AVG-16 [12], MDG2.1 [13], PGC-2 [14], and JC-410 [15], were unable to express follicular stimulating hormone receptor (FSHR), luteinizing hormone receptor (LHR) [10], or cytochrome P450 family 19 subfamily a member 1 (CYP19A1) [15], which are key genes for granulosa function. Additionally, other GC line such as JC-410 could only produce a very low level of progesterone [10], while PGV lost the ability to produce progesterone [10]. Thus, a GC line that more closely resembles the primary GCs would be desirable for studying the function and regulation of folliculogenesis [16].

To address the issue regarding the loss of physiological function of transfected cells established via constitutive expression of Large T or Tert after long-term culture [16,17], a reversible cell line approach has recently received a great deal of interest for the establishment of multiple cell lines from other somatic tissues [18,19,20,21]. More functional phenotypes of the immortal cells might be recovered by turning off the expression of Large T or Tert using the Cre/LoxP-based inducible system [17]. Thus, a reversible immortalized GC line may be desirable for studying their function in vitro. The tetracycline (Tet)-on 3G system, otherwise, may provide an alternative effective inducible platform to regulate gene expression in many cells from human and animals [22]. Therefore, this study was carried out to establish a reversible porcine GCs line by turning on/off the expression of Large T using the Tet on-3G system, and investigated whether the established GCs line might have the biological function of primary GCs. Here, we report the generation of a reversible porcine GC line, termed as conditional immortal porcine GC (CIPGC), which is capable of estradiol production in response to gonadotrophins, offering a unique in vitro model for studying the functional regulation of these important cells during ovarian follicular development.

## 2. Materials and Methods

### 2.1. Construct of the Inducible and Reversible Large T Lentiviral Plasmid

The inducible and reversible Large T lentiviral plasmid was constructed by remodeling the Tet-on 3G lentiviral vector (Addgene, #50661, Cambridge, MA, USA). Tet-on 3G lentiviral plasmid was integrated with inducible expression of Large T and mCherry gene, and manipulated by tetracycline (Tet)-controlled transcriptional activation. In addition, the puromycin resistance gene (Puro) was used for cell screening. The tetracycline inducible (Tet-on) lentiviral expression vectors with TRE 3G promoter bound to rrTA and doxycycline (Dox, a more stable tetracycline analogue, Sigma, D9891, St. Louis, MO, USA) were used in this study. These vectors were applied to control the expression of the gene of Large T and reporter gene of mCherry, and jointed by Thosea asigna virus 2A self-cleaving peptides (T2A) sequence.

### 2.2. Isolation and Cultivation of Granulosa Cells

Pig ovaries were collected from a local commercial slaughterhouse and transported to the lab in warm sterile saline (38 °C) within 2 h. Primary granulosa cells (GCs) were isolated using 10 mL syringe from 2–3 mm ovarian follicles followed by three washes of warm sterile saline. Then, the primary GCs without cumulus–oocyte complexes (COCs) were collected and then subjected to low-speed centrifugation at 1000 rpm for 5 min, followed by three washes with PBS to get rid of white blood cells. The primary GCs were then transferred to 10 cm culture dish for in vitro culture with DMEM medium (11960044, Gibco, Grand Island, NE, USA) supplemented with 10% fetal bovine serum (FBS, Gibco, 10099-141), 1% GlutaMAX™ supplement (Gibco, 35050061), and 20 ng/mL epidermal growth factor (EGF, AF-100-15, Peprotech, Rocky Hill, CT, USA). On the second day of isolation, the primary GCs were changed culture medium, followed by three washes with PBS to remove the remaining unattached white blood cells.

### 2.3. Lentivirus Packaging, Infection, and Cell Screening

The lentiviral gene transfer plasmids of 10 µg pLVX-Tet3G-Large T-T2A-mCherry-Puror, as well as the package plasmids of 10.4 µg psPAX2 and 3.5 μg pMD2.G, were transfected with 70% confluent 10 cm plates of 293FT cells (ATCC, EY-X0869, Rockefeller, MD, USA) by polyfect transfection reagent (301105, Qiagen, Hilden, Germany). The recombinant lentiviral particles found in the supernatant were collected at 48 and 72 h, followed by filtration (0.45 µm) into sterilized 50 mL centrifuge tubes. The high-concentration lentivirus was harvested through an ultrafiltration device (Millipore, UFC9011096, Germany) by centrifuging at 4000 rpm, 4 °C for 30 min, and lentivirus aliquots were stored at −80 °C until further use. Viral particles were then suspended in 500 µL GC complete medium with 6 µg/mL polybrene (Sigma, H9268), and lentiviral solution at a multiplicity of infection (MOI) of 20 was applied to infect GCs after first passage for 6 h. Infected GCs were continued to culture for another 48 h, followed by 14-day screening with 1 µg/mL puromycin (Gibco, A1113803) and induction of 50 ng/mL Dox. The infection results were analyzed by observing mCherry expression under a fluorescent microscope (IX73, Olympus, Tokyo, Japan).

The CIPGCs were cultured in DMEM supplemented with 10% FBS, 1% GlutaMAX™, and 50 ng/mL Dox, and maintained long-term propagation for more than six months with consecutive passages. This long-term cultured CIPGCs were treated with or without Dox for six days, and then used for the determinations of cell cycle, cell proliferation, immunofluorescence, mRNA expression, and steroid hormone production.

### 2.4. Cell Cycle Analysis

Cell cycle analysis were conducted to compare the proliferative ability of CIPGCs when treated with or without Dox. The CIPGCs with and without Dox induction for 2, 4, and 6 d were harvested, respectively, and fixed with 70% ice-cold ethanol at 4 °C overnight, centrifuged at 1500 rpm for 5 min, and then resuspended in PBS. Subsequently, the cells were stained with 50 µg/mL propidium iodide (CF0032, Beijing Dingguo Changsheng Biotechnology Co, Beijing, China), 100 µg/mL RNase A (9901-99-4, Genview, Galveston, TX, USA), and 0.2% Triton X-100 for 30 min in the dark at room temperature. Cell cycle analysis was conducted using a FACSAria II system (BD Biosciences, Franklin Lakes, NJ, USA).

### 2.5. Cell Proliferation Assays

The long-term cultured CIPGCs after treatment with or without Dox for six days were seeded into the 96-well plates at a cell density of 1 × 10^3^ per well, and were examined for cell proliferation via the CellTiter 96^®^ AQueous One Solution Cell Proliferation Assay (MTS; G3582, Promega, Madison, WI, USA). Briefly, 20 µL of CellTiter 96^®^ AQueous reagent was added into each well of the 96-well assay plate containing sample and 100 µL of culture medium. The plate was incubated at 37 °C for 1 h in a humidified atmosphere with 5% CO_2_, and was recorded under the absorbance at 490 nm using a 96-well plate reader using an ELISA machine.

### 2.6. Immunofluorescence

After six days of Dox induction, immunofluorescent staining was performed [23], and the CIPGCs were treated with 1% bovine serum albumin (BSA, Sigma-HZB0148) and 0.01% Triton-100 (AR-0341, Beijing Dingguo Changsheng Biotechnology Co). Then, the CIPGCs were treated with primary antibodies (Rabbit anti-FSHR, Absin-abs120271, 1:200 dilution, Univ-bio, Shanghai, China; Rabbit anti-CYP11A1, AB2060, 1:200 dilution, Liankebio, Hangzhou, China; Rabbit anti-3β-HSD, ab150384, 1:200 dilution, Abcam, Cambridge, UK, USA) at 4 °C overnight. After washing three times with PBS for 5 min, CIPGCs were incubated for 1 h with secondary antibodies (Alexa Fluor^®^ 488 Goat Anti-Rabbit IgG, Invitrogen, A11034 and Alexa Fluor^®^ 568 Goat Anti-Rabbit IgG, Invitrogen, A11036, all for 1:500 dilution, Camarillo, CA, USA) at room temperature. Nuclei were counterstained with 10 µg/mL Hochest33342 (Molecular Probes, H3570, Waltham, MA, USA) for 10 min. Fluorescence signals were detected using the fluorescent microscope.

### 2.7. RNA Extraction and Quantitative Reverse Transcriptase (RT)-PCR

In order to compare the relative mRNA expression of specific genes in long-term cultured CIPGCs after treatment with or without Dox for six days, total RNA was extracted from CIPGCs in both groups using the RNeasy Mini Kit (Qiagen, Hilden, Germany) according to the manufacturer’s instructions. The cDNA was synthesized from 1 μg RNA using Superscript cDNA Reverse Transcription Kit (Invitrogen, Camarillo, CA, USA). Quantitative RT-PCR was performed using SYBR Green PCR Master Mix (Bio-Rad, Hercules, CA, USA) in an Applied CFX Connext^TM^ Real-time PCR machine. The primer sequences of targeted genes (*LHR*, luteinizing hormone receptor; *CYP11A1*, cytochrome P450 family 11 subfamily a member 1; *3β-HSD*, 3β-hydroxysteroid dehydrogenase; *StAR*, steroidogenic acute regulatory protein; *CYP19A1*, cytochrome P450 family 19 subfamily a member 1; *LIFR*, leukemia inhibitory factor receptor; *PCNA*, proliferating cell nuclear antigen; *CCNB1*, cyclin B1) are given in Supplementary Table S1. The mRNA expression of targeted genes was normalized to that of house keeping gene *GAPDH*.

### 2.8. Morphology and Phentypic Analysis of CIPGCs with or without Dox

In order to investigate the efficiency of CIPGCs to be reversible controlled by Dox withdrawal, we cultured the long-term propagated CIPGCs with DMEM, 10% FBS, and 1% GlutaMAX™ but no Dox for 14 days, when most cells became ageing and dead. On day 15, addition with 50 ng/mL Dox was re-introduced in the culture system for another nine days, when the CIPGCs reached almost confluency. Morphology changes along with mCherry expression were observed under fluorescent microscope accordingly.

### 2.9. Determination of Steroid Hormones’ Secretion

The supernatants of the CIPGCs with or without Dox induction were collected at 2, 4, and 6 d. The levels of estradiol and progesterone in the supernatant were detected by estradiol ELISA Kit (No. 582251, Cayman, Arbor, MI, USA) and progesterone ELISA Kit (No. 582601, Cayman), following the protocols of the manufacturers, respectively.

In order to further compare the steroid hormone productions of the CIPGCs with those of primary GCs, the cell supernatants were harvested after treatments with 0.01 U/mL porcine FSH (Sioux Biochemicals, Sioux Center, IA, USA) [24] or 0.02 U/mL LH (Sioux Biochemicals) [25] for 24 h, and then the levels of estradiol were determined as mentioned above.

### 2.10. Statistics Analysis

Data analysis was performed using Student’s *t*-test in the GraphPad software (GraphPad Software, La Jolla, CA, USA) to evaluate the statistical differences of groups. Student’s *t*-test was used for the comparison of results between two groups [Dox (+) vs. Dox (–)]. One-way analysis of variance (ANOVA) with Duncan’s multiple comparison was employed for the comparison of data among three groups. The results were presented as the means ± standard error (*n* ≥ 3). *p* <0.05 was indicated as a statistically significant difference. All the experiments were repeated at least three times, except that the immunofluorescent analysis was repeated twice.

## 3. Results

### 3.1. Construction of the Inducible Large T Expressing Lentiviral Plasmid

**Figure 1:**
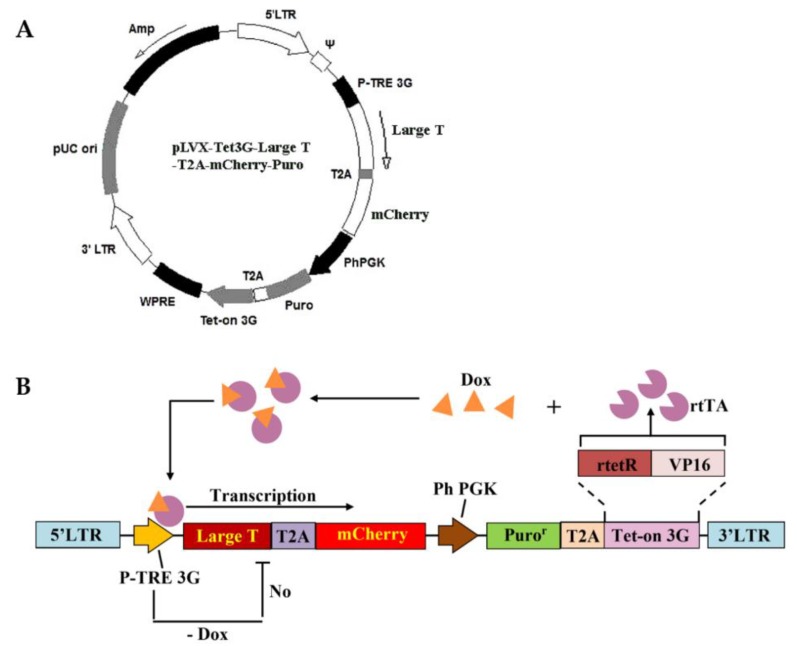
Lentiviral plasmid design and construction. (**A**) The lentiviral plasmid map; (**B**) the elements the of constructed plasmid: LTR, long terminal repeats sequence; P-TRE 3G, third generation Tet-inducible promoter; Large T, Simian vacuolating virus 40 Large T antigen; T2A, 2A self-cleaving peptides; mCherry, a red fluorescent protein; Ph PGK, human phosphoglycerate kinase 1 promoter; Puro^r^, puromycin resistance gene; Tet-on 3G (rtTA), third-generation doxycycline-responsive transactivator protein.

A lentivirus-based inducible Large T and mCherry expression vector was constructed. The inducible expression was achieved using the Tet-on 3G system, which linked Large T and mCherry coding sequence via T2A sequence, allowing simultaneous expression of both of the Large T and red florescence proteins (Figure 1). Puromycin was used as a selection antibiotic marker to achieve stable vector integration.

### 3.2. Isolation and Lentivirus Transduction of Porcine GCs

**Figure 2:**
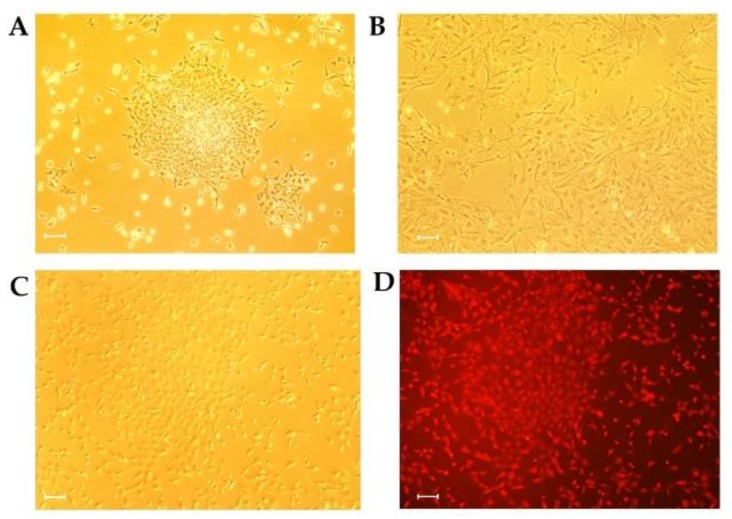
Isolation of granulosa cells (GCs) and transfection with Tet-on 3G Large T lentivirus. (**A**) The primary GCs were isolated from the ovary tissue and cultured at day 1. (**B**) The primary culture GCs were differentiated on the fifth day. (**C**,**D**) GCs with expression of Large T and mCherry maintained long-term proliferation as primary culture GCs. Bar at 50 μm.

Porcine primary GCs isolated from ovarian follicles showed a small and fibroblast-like morphology under primary culture (Figure 2A). After five days, these GCs stopped proliferating and appeared to be larger and longer; a morphology that is consistent with differentiative GCs (Figure 2B). Porcine primary GCs were transduced with lentivirus harboring the Tet-on-Large T-T2A- mCherry gene. Upon induction of Large T expression with Dox, this transduced GC line, named conditional immortal porcine GC (CIPGCs), displayed proliferation morphology of small and non-stretched cells, and expressed mCherry fluorescence. In the presence of puromycin selection, the induced stable Large T expressed GCs steadily proliferated and passaged in vitro for at least six months (Figure 2C,D; Supplementary Figure S1).

### 3.3. Large T-T2A-mCherry Expression Is Reversible in CIPGCs

**Figure 3:**
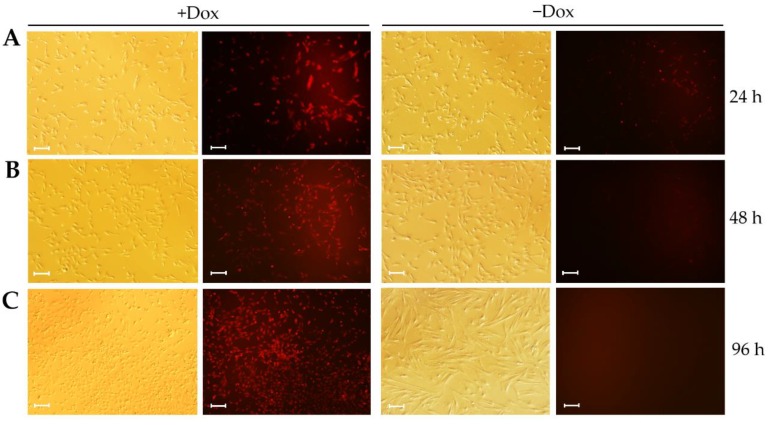
Dox inducible expression of Large T and mCherry. The mCherry expression was observed in CIPGCs with or without Dox at 24 h (**A**), 48 h (**B**), and 96 (**C**), respectively. CIPGCs, conditional immortal porcine GCs. The experiment was repeated three times. Bar at 50 μm.

To confirm reversible Large T-T2A-mCherry expression in CIPGCs upon the removal of Dox from culture, we determined the expression of mCherry under a fluorescent microscope. It was found that the expression of mCherry began to decrease 24 h after Dox withdrawal (Figure 3A), and then gradually disappeared by 48 h (Figure 3B) and 96 h (Figure 3C). This was accompanied by the gradual elongation of the granulosa cells (Figure 3; −Dox). In contrast, the CIPGCs cultured in the presence of Dox continuously expressed mCherry and maintained the appearance of proliferative primary cultured GCs (Figure 3, +Dox). These results suggest that Large T expression was responsive to Dox in a time-dependent manner in CIPGCs.

### 3.4. The Proliferation of CIPGCs Is Controlled by Dox Induction

**Figure 4:**
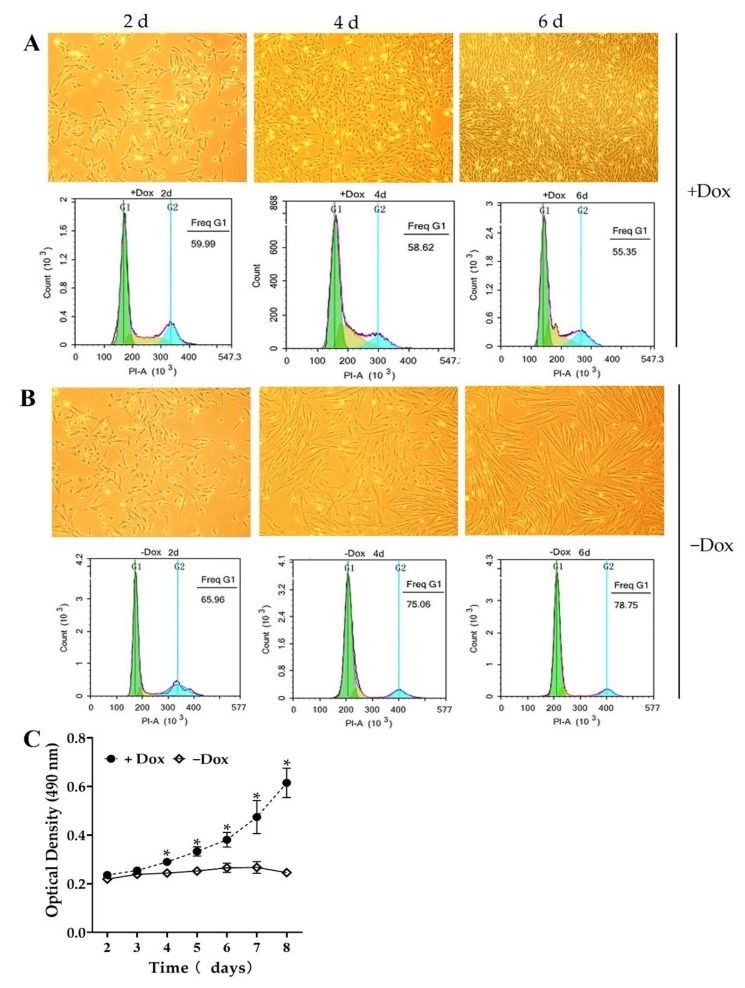
Proliferation of CIPGCs induced by Dox. (**A**) The morphology and cell cycle analysis of CIPGCs with Dox induction for culture. (**B**) The morphology and cycle analysis of CIPGCs without Dox induction for culture, cell cycle analysis showed that more cells were in S/M (Synthesis/Mitotic) phase during induction by Dox; at the withdrawal of Dox, percentage of cells in S/M phases decreased. (**C**) Cell proliferation curve during an eight days’ culture in the presence and absence of Dox. Data are represented as mean ± SEM of three independent experiments (*n* = 6). * stands for significant difference between CIPGCs +Dox and –Dox group (*p* < 0.05). Bar at 50 μm.

In the presence of Dox, the CIPGC line showed proliferative morphology, in which less than 60% of the cells were at G1 phase of the cell cycle when measured via flow cytometry (Figure 4A). However, in the absence of Dox, CIPGCs demonstrated gradual elongation, and G1 phase cell percentage increased to 79% by day 6 of Dox withdrawal (Figure 4B). Consistently, cell proliferation assay revealed that, in the presence of Dox, CIPGCs grew rapidly, while they stopped proliferating in the absence of Dox (Figure 4C). These data demonstrated the distinct proliferation potential and status of CIPGCs in the absence and presence of Dox, respectively.

**Figure 5:**
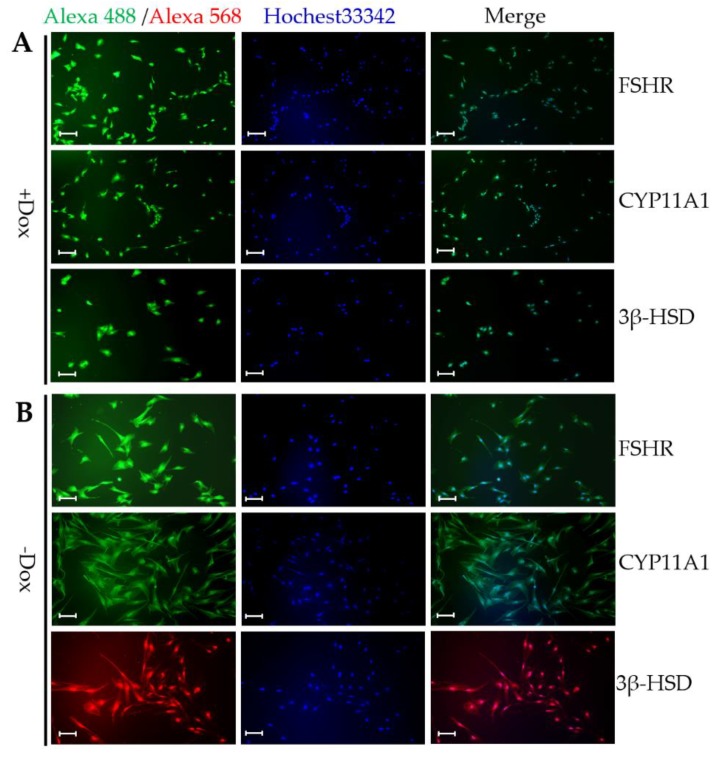
Expression of granulosa cell markers in the CIPGC line by immunofluorescent staining in the presence and absence of Dox. Immunofluorecent staining of FSHR, CYP11A1, and 3β-HSD in CIPGCs with (**A**) and without (**B**) Dox induction. CYP11A1, cytochrome progesterone 50 family 11 subfamily A member 1; 3β-HSD, 3β-hydroxysteroid dehydrogenase; FSHR, follicular stimulating hormone receptor. The experiment was repeated twice. Bar at 50 μm.

Next, we further investigated if the CIPGCs expressed markers that are consistent with primary GCs via immunofluorescent analysis. As shown in Figure 5, follicular stimulating hormone receptor (FSHR) and enzymes involved in steroid synthesis and metabolism, such as Cytochrome progesterone 50 family 11 subfamily A member 1 (CYP11A1) and 3β-hydroxysteroid dehydrogenase (3β-HSD), are expressed in CIPGCs cultures in both the presence (Figure 5A) and absence of Dox (Figure 5B) after six days. However, the morphologies of these two groups of GCs were distinguishable from each other in that they were larger, and the elongated CIPGCs are apparent in the Dox (–) group.

**Figure 6:**
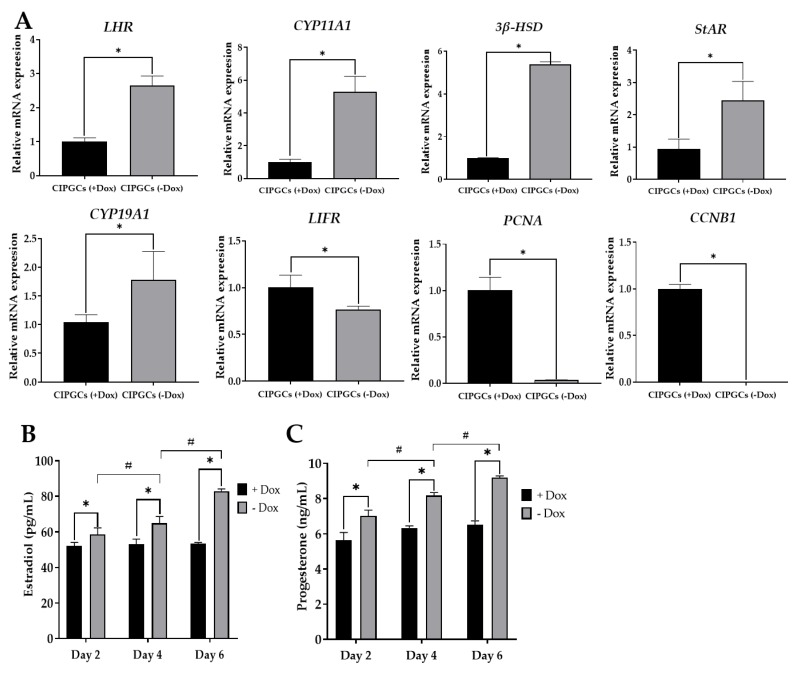
Expression of granulosa markers in the GC line when cultured in the presence and absence of Dox. (**A**) The mRNA expression of *LHR*, *CYP11A1*, *3β-HSD*, *StAR*, *LIFR*, *PCNA*, and *CCNB1* by quantitative PCR after culturing for six days with or without Dox. Estradiol (**B**) and progesterone (**C**) detection by ELISA detection (*n* = 6). *LHR*, luteinizing hormone receptor *CYP11A1*, cytochrome P450 family 11 subfamily A member 1; *3β-HSD*, 3β-hydroxysteroid dehydrogenase; *StAR*, steroidogenic acute regulatory protein; *CYP19A1*, cytochrome P450 family 19 subfamily a member 1; *LIFR*, leukemia inhibitory factor receptor; *PCNA*, proliferating cell nuclear antigen; *CCNB1*, cyclin B1; FSH, follicular stimulating hormone; LH, luteinizing hormone. The experiment was repeated three times. * stands for significant difference between +Dox and –Dox group at specific day (*p* < 0.05). ^#^ stands for significant difference from three time points in the –Dox group (*p* < 0.05).

Quantitative PCR analysis further confirmed the expression of these GC markers, and revealed that the mRNA expression levels of *LHR* (luteinizing hormone receptor), *CYP11A1* (cytochrome P450 family 11 subfamily A member 1), *3β-HSD* (3β-hydroxysteroid dehydrogenase), *StAR* (steroidogenic acute regulatory protein), and *CYP19A1* (cytochrome P450 family 19 subfamily a member 1) were higher in the Dox (–) group compared with that of the Dox (+) group, while the mRNA expression of genes related in cell proliferation genes like *LIFR* (leukemia inhibitory factor receptor), *PCNA* (proliferating cell nuclear antigen), and *CCNB1* (cyclin B1) was lower in the Dox (–) group (Figure 6A).

We next investigated the levels of estradiol and progesterone production by CIPGCs in the presence and absence of Dox. It was found that the levels of both estradiol and progesterone were significantly higher in the Dox (–) group compared with those with Dox addition (Figure 6B,C). Moreover, while the levels of the two hormones remain similar in the presence of Dox, both estradiol and progesterone showed a time-dependent increase during the six days of Dox withdrawal. This finding further confirms that GCs from the Dox (–) group are more differentiated than those from the Dox (+) group.

### 3.5. The Reversible Regulation on Proliferation State of CIPGCs by Dox

**Figure 7:**
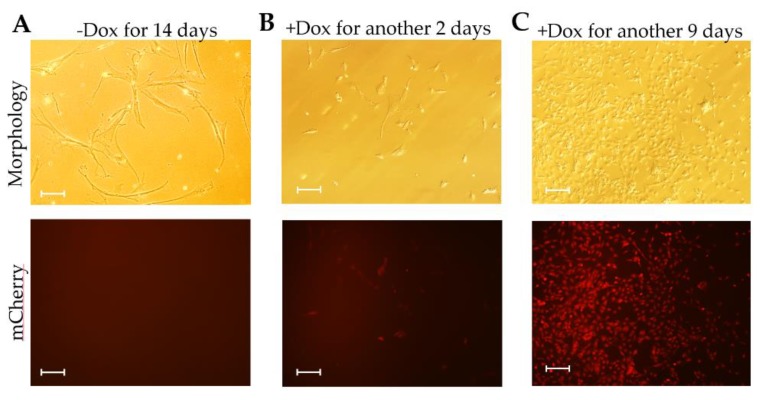
Morphology and phenotypic analysis of the CIPGCs after Dox withdrawal followed by Dox induction. (**A**) The CIPGCs became ageing after culturing without dox for 14 days and had no expression of mCherry. The ageing CIPGCs were then re-treated with dox induction for another two days (**B**) or nine days (**C**), and the mCherry expression was recovered. Bar at 50 μm.

Withdrawal of Dox for 14 days resulted in the ceasing of proliferation, cell elongation, senescence, and absence of mCherry expression (Figure 7A). Interestingly, this morphology of the CIPGCs could be reversed by the addition of Dox. Two days after Dox supplementation, the CIPGCs shortened and started expressing Large T, as reflected by mCherry fluorescence (Figure 7B). In addition, the CIPGCs became much smaller in size, and more proliferative at day 14 of Dox culture (Figure 7C). These results suggest that the induced Large T expression reversed the ageing CIPGCs to proliferative and active CIPGCs.

### 3.6. Comparison of Steroidogenesis and Gonadotropin Response of CIPGCs and Primary GCs

**Figure 8:**
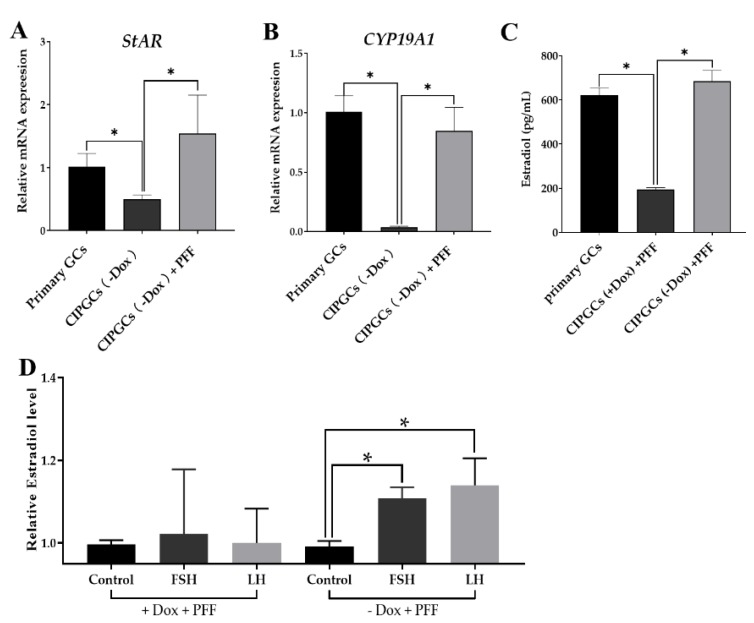
The function analysis of the CIPGCs. The mRNA expression of *StAR* (**A**) and *CYP19A1* (**B**) by quantitative PCR after culturing for six days without Dox treatment in company with or without PFF when compared with primary GCs. (**C**) Determination of estradiol level among primary GCs, CIPGCs (+Dox), and CIPGCs (–Dox). (D) The estradiol level in the CIPGCs (–Dox) with addition of FSH and LH. PFF, porcine follicular fluid. Data are represented as mean ± SEM of three independent experiment (*n* = 3). * stands for significant difference between two groups (*P* < 0.05).

We next further examined the steroidogenesis potential and CIPGC in comparison with freshly isolated primary GC. The quantitative PCR analysis showed that the mRNA expression of hormone synthesis-related enzymes was not significant between primary GCs and GIPGCs (–Dox) + porcine follicular fluid (PFF) groups. As shown in Figure 8A,B, for the expression of key estradiol synthesis enzymes (*CYP19A1* and *StAR*), CIPGCs without Dox addition were significantly lower than that in primary GCs (Figure 8A,B). We hypothesized that this difference may be because of the lack of exposure of the CIPGCs to the porcine follicular fluid (PFF), in which GCs are physiologically bathed. To test this hypothesis, we primed the CIPGCs with PFF. As shown in Figure 8A,B, the mRNA levels of these two key steroidogenesis enzymes are the same between CIPGCs and the primary GCs. As to be expected, priming of CIPGCs with PFF also allows the granulosa cell line to produce estradiol to the level that is the same as that of the primary GC. Moreover, PFF priming also resulted in the estradiol production of CIPGCs Dox (–) to the level that is similar to that of primary GC, although the estradiol level in the CIPGCs Dox (+) is significantly lower (Figure 8C). Interestingly, PFF priming of CIPGCs in Dox (–) group also enabled the cell line to respond to FSH and LH in estradiol production, while CIPGCs upon Dox exposure could not increase the estradiol level under FSH and LH stimulation (Figure 8D).

## 4. Discussion

The previously common method for cell immortalization is to introduce the simian virus 40 (SV40) T antigen into target primary cells. The large T antigen forms complexes with tumor suppressors, such as pRB-1 and p53, and induces DNA synthesis in both dividing and quiescent cells, leading to extension of the lifespan of the cells [26]. This approach has been used for transformation of various primary cell types [27,28,29]. One of the major limitations of permanent immortal cell lines is their proliferative status, which prevents the development of the full physiological phenotype [30]. This may complicate the interpretation of data obtained from these cell lines. To overcome this problem, conditional immortalization is desirable. Using this approach, inducible immortal astrocytes [31], adipocyte precursor cells [32], and hematopoietic precursor cells [33] have all been successfully established. However, an inducible immortal GC line has not been established.

In this study, we constructed a Large T expressing lentiviral vector driven by the tetracycline-inducible (Tet-on) system, which can switch on and off the expression of the gene, and thus regulate the proliferation of the transformed GCs in vitro. The Tet-on system has been extensively used for reversible induction of gene expression in many mammalian cells and transgenic animals [34,35,36,37,38]. This Tet-on 3G system is based upon the interaction between a reverse transactivator (rtTA) and the tetracycline-responsive element (TRE), which is composed of seven copies of the prokaryotic tetracycline operator site (tetO) fusing with a minimal CMV promoter. In the presence of tetracycline or its derivate doxycycline (Dox), the rtTA transactivator activates minimal promoters and the downstream of an array of tetO sequences, allowing the expression of the transgene [39]. The Tet-on 3G system has been claimed to possess 10–100 times higher sensitivities to Dox than the traditional Tet-on system [40]. With the inducible promoter based on the Tet-on 3G and the Tet-on 3G-response element (TRE3G) [41], our Large T antigen is co-expressed with mCherry (as a reporter). Our data suggest that this inducible system works efficiently in the transduced GC (CIPGCs). In the presence of Dox, CIPGC steadily expressed Large T, having similar morphologic characteristics as primary GCs. In contrast, Large T expression was undetectable in the absence of Dox after 48 h.

Our data revealed that CIPGCs exhibited the functional characteristics of primary GCs such as the expression of steroid enzymes, as well as the ability for estradiol and progesterone production. In the presence of Dox, CIPGCs were capable of continued proliferation for at least six months. Moreover, at Dox withdrawal, their cell cycle shifted toward G1 phase and gradually lost replicative ability. These CIPGCs demonstrated increased expression of LHR and enzymes such as CYP11A1, 3β-HSD, StAR, and CYP19A1, all of which are crucial for steroid hormone production [42,43], as well as increased production of estradiol and progesterone. These data suggest that CIPGCs are capable of reversible immortalization and reulating steroidogenesis. Interestingly, upon a repeated supplementation with Dox for two days, our CIPGCs returned to the original proliferative morphology, further demonstrating the efficiency of controlled immortalization.

Although the mRNA levels of StAR and CYP19A1 were significantly lower in the CPIGCs compared with that of the freshly isolated primary GCs, the mRNA expression of these steroidogenesis genes is enhanced to the levels that are comparable to those of primary GCs, when they are cultured in the presence of PFF. This finding suggests that our CIPGCs retain the ability to function similar to primary GCs, when they are placed in an environment exposed to PFF. Our finding that estradiol production responded to FSH and LH stimulation in the presence of PFF further supports this notion. It showed that this cell line established might be inducible, stably proliferate, and reversely display the physiological function of primary cells.

In conclusion, we have successfully established an inducible porcine GC line (CIPGCs) via the Tet-on 3G system. Our established CIPGCs retain the ability of maintaining physiological functions, and may provide a unique valuable model for future research on the specific gene regulatory mechanisms of this important ovarian somatic cell.

## Supplementary Materials

The following are available online at https://www.mdpi.com/2073-4409/9/1/156/s1, Table S1: Primer sequence, and accession number of target genes. Figure S1: Long-term cultivation and screening of the CIPGCs.
