# Supplementary material for: Establishment of A Reversibly Inducible Porcine Granulosa Cell Line
Source: Cells. 2020 Jan 8;9(1):156. doi: 10.3390/cells9010156 (PMC7017277; doi:10.3390/cells9010156)
Supplement: Supplementary file 1 [file cells-09-00156-s001.pdf]

Article

Establishment of A Reversibly Inducible Porcine Granulosa Cell Line

Yinshan Bai [1,2], Cui Zhu [1], Meiying Feng [3], Bo Pan [2], Shouquan Zhang [3], Xiaoshu Zhan [2], Huifang Chen [1], Bingyun Wang [1], and Julang Li [2,*]

[1] School of Life Science and Engineering, Foshan University, Foshan 528231, China; xuefei200403@163.com (Y.B.); juncy2010@gmail.com (C.Z.); chenhuifang07@163.com (H.C.); bywang63@163.com (B.W.)
[2] Department of Animal Biosciences, University of Guelph, Guelph, ON N1G 2W1, Canada; bopan@uoguelph.ca (B.P.); xzhan01@uoguelph.ca (X.Z.)
[3] College of Animal Science, South China Agricultural University, Guangzhou 510642, China; jony.ya@163.com (M.F.); sqzhang@scau.edu.cn (S.Z.)
* Correspondence: jli@uoguelph.ca; Tel.: +519-824-4120 (ext. 52713)

Supplementary Table S1. Primer sequence, and accession number of target genes.

Gene		Primer sequence (5′-3′)	Product size (bp)	Accession No.
GAPDH	F:	CGTGTCGGTTGTGGATCTGA	260	XR_002343817
	R:	CAGTCTTGGTCAGTGGGGTC		
LHR	F:	TGCTTTCCAAGGGATGAATAACG	279	JN120794
	R:	ATCCTATTCTCTGAAAAAACTGCC		
CYP11A1	F:	TTCCAGAAGTATGGTCCCATTTA	501	NM_214427
	R:	TGAGCATGGGGACACTAGTGTGG		
3β-HSD	F:	CCTTCAATCGCCACTTCG	157	AF232699
	R:	TCCTTGTGCTGCTTCACCA		
StAR	F:	GGAGAGCCGGCAGGAGAATG	183	NM_213755.2
	R:	CTTCTGCAGGATCTTGATCTTCTTG		
CYP19A1	F:	GGGTCACAACAAGACAGGACT	202	NM_214429
	R:	ACCTGGTATTGAAGATGTGTTTTT		
LIFR	F:	CAAGACCGTGCGTTGAGC	196	AM268512
	R:	GAGTAACTGTCCCTGTAAGAATCCT		
PCNA	F:	GCAGAGCATGGACTCGTCTC	120	NM_001291925
	R:	TTGGACATGCTGGTGAGGTT		
CCNB1	F:	CCAACTGGTTGGTGTCACTG	148	NM_001170768
	R:	GCTCTCCGAAGAAAATGCAG		

GAPDH, glyceraldehyde-3-phosphate dehydrogenase; *LHR*, luteinizing hormone receptor; *CYP11A1*, cytochrome P450 family 11 subfamily A member 1; *3β-HSD*, 3β-hydroxysteroid dehydrogenase; *StAR*, steroidogenic acute regulatory protein; *CYP19A1*, cytochrome P450 family 19 subfamily a member 1; *LIFR*, leukemia inhibitory factor receptor; *PCNA*, proliferating cell nuclear antigen; *CCNB1*, cyclin B1.

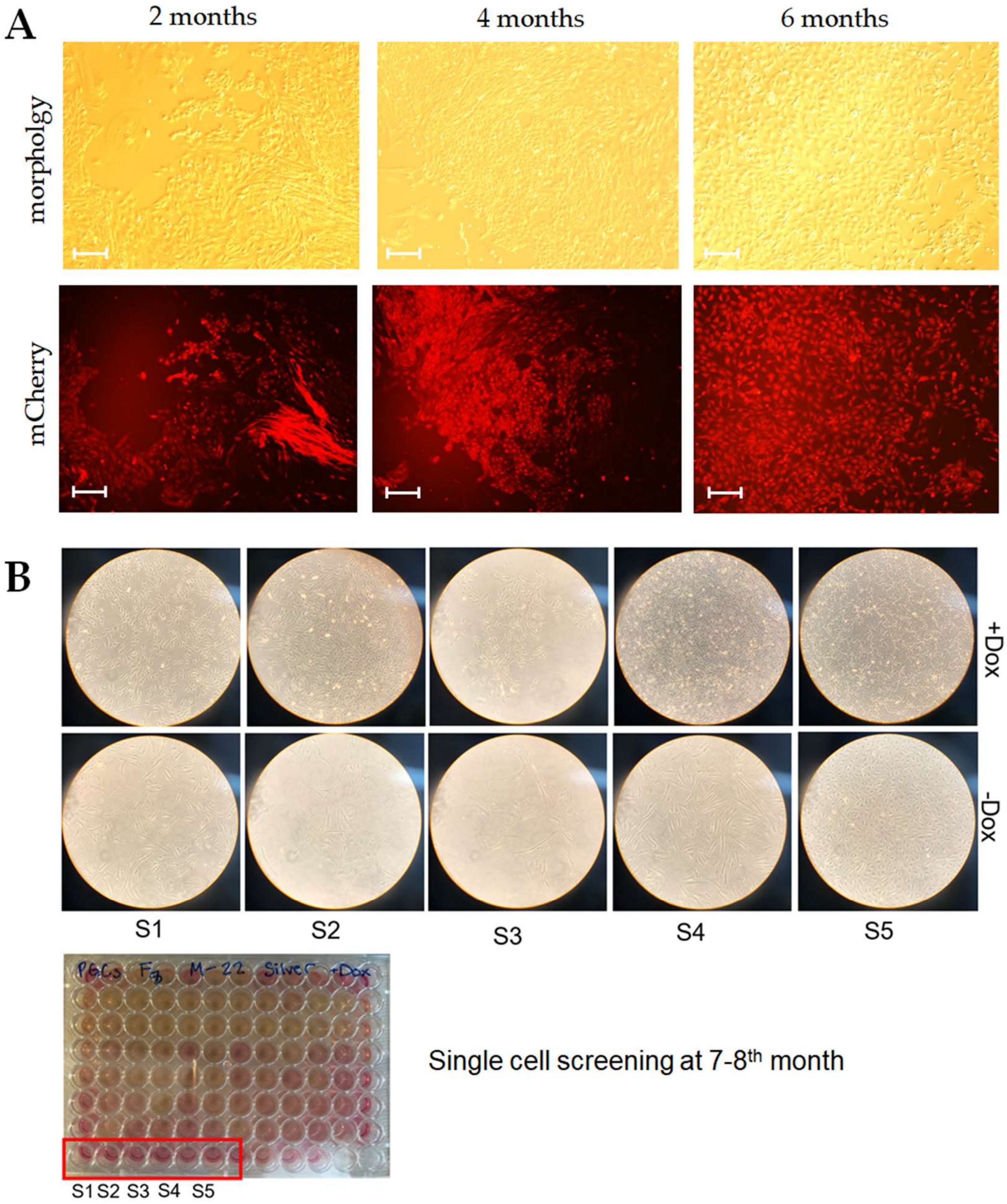

Supplementary Figure S1. Long-term cultivation and screening of the CIPGCs. **(A)** The long-term propagation of the CIPGCs. After transfecting with Tet-on-Large T, the GCs were screened with puromycin for consistent morphology and stable mCherry expression, and passaged *in vitro* for at least six months to establish the CIPGCs. The representive pictures of cell morphology and mCherry expression in the CIPGCs at the 2nd, 4th, and 6th months were shown. **(B)** Single cell screening of the CIPGCs at about 7-8 months after transfection with Tet-on-Large T. Five single cell lines (S1, S2, S3, S4, and S5) from the long-termed cultured CIPGCs were harvested and maintained steady propagation with 50 ng/mL Dox, while Dox withdrawal induced loss of proliferation and became ageing for these cells. Bar at 50 μm.
